# The Decline of Life in Health and Disease
*On the Decline of Life in Health and Disease; being an Attempt to investigate the Causes of Longevity, &c. By Barnard Van Oven, M.D. London: Churchill, 1853.


**Published:** 1853-10-01

**Authors:** 


### THE DECLINE OF LIFE IN HEALTH AND DISEASE.*

The important subject of longevity has been but little investigated in this country. It cannot, therefore, be otherwise than gratifying to our feelings, to find that a gentleman of admitted scientific and practical eminence has been, for a series of years, devoting his mind to the study of the art of prolonging life. Are we not, as a body of professional men, too much disposed to neglect the subject of *hygiène?* and are we sufficiently alive to the importance of studying with care and attention the causes that shorten or promote longevity? That we have the power of prolonging life much beyond the scriptural limits by the adoption of judicious hygienic measures, is an indisputable fact; and he who is able to suggest the best means for accomplishing so beneficial a result, is entitled to our warmest gratitude. Dr. Van Oven's work is replete with valuable data, having a direct and important bearing upon the subject of longevity. It is written with elegance and good taste. The rules laid down by the author for the prolongation of human life are based upon an accurate knowledge of the principles of medicine, and an enlarged experience of the treatment of disease at the bed-side.

We subjoin the following observations relative to a point of psychological interest:—

"When we speak, then, of the growth of the mind, we do not mean the growth of the actual mental powers, but the augmentation of the stores of knowledge which are gradually acquired by the mind, and which are essential to its daily action. Now these stores of knowledge are gained only through the organs of sensation, and it is therefore evident that the perfection of these organs is necessary for supplying the mind with wholesome food; to promote, not the growth of its abstract power (which, being incorporeal, may be always equal), but the accumulation of those clear impressions from without, which enable it so to exert itself as to act in that manner which we call *wisely*. The organs of infancy cannot convey correct impressions to the mind, nor is the brain, perhaps, sufficiently consolidated to receive them properly; it is neces-

* On the Decline of Life in Health and Disease; being an Attempt to investigate the Causes of Longevity, &c. By Barnard Van Oven, M.D. London: Churchill, 1853.

sary that a certain maturity of organization should exist, in order that the impression conveyed to the mind be clear and definite, that many various and repeated impressions should have been made before it can have gained an adequate store of knowledge, an adequate mass of materials whereon to exert its powers. Perception, memory, imagination, and judgment, are usually regarded as the four great powers of the mind. Of these perception is the great supplying power on which the others depend. The eye perceives all that passes around it; and by the use of letters it is enabled to peruse the records of past events, and thus, as it were, to see all that *has ever* been, and all that *now is*, throughout the world. Nay, more, to commune with the bygone races of mankind, and learn all that they saw, heard, read, or thought. In the same way the ear, the touch, the taste, the smell, minister to the mental storehouse, carrying valuable impressions to all around. Memory is but the power of calling up, from the well-arranged stores of knowledge, past impressions, and the effects produced by them."

We would direct the particular attention of our readers to the chapter on apoplexy and hemiplegia. It contains some valuable suggestive matter.

Dr. Van Oven concludes his very interesting and important work with the following sensible remarks:—

"If a complete system of public hygiène were established, pestilential and epidemic diseases would cease to appear; a wise physical as well as moral training in early youth would be universal, and would gradually eradicate hereditary faults of organization, and thus, by a combination of wise legislation on the part of the government, and of prudence and obedience on the part of the people, the nation might be rendered more healthful, more vigorous, more virtuous, more happy. Oh, may this soon be! May this great country, distinguished as it is in arms and arts, the queen of commerce, the home of freedom, the refuge of the oppressed, become remarkable for healthfulness, even in its crowded cities and manufacturing towns! May her inhabitants be distinguished alike by the perfection of their physical development, the completeness of their mental powers, and the purity of their moral conduct—a model for the admiration and imitation of mankind—a free, a healthful, and a happy people!"

To this we say, Amen. We sincerely trust that the author of this able and philosophical work will continue his investigations into this much neglected branch of medical literature. His work should be studied by non-professional as well as by professional readers.

